# Consensus-based recommendations for psychosocial support measures for parents and adult children at the end of life: results of a Delphi study in Germany

**DOI:** 10.1007/s00520-021-06452-x

**Published:** 2021-08-07

**Authors:** Franziska A. Herbst, Laura Gawinski, Nils Schneider, Stephanie Stiel

**Affiliations:** Institute for General Practice and Palliative Care, Hannover Medical School, Carl-Neuberg-Strasse 1, 30625 Hannover, Germany

**Keywords:** Palliative care, Adult children, Parents, Parent–child relations, Delphi technique, Consensus

## Abstract

**Purpose:**

The availability of psychosocial support measures has a significant impact on the quality of life of terminally ill and dying patients and the burden experienced by their relatives. To date, no intervention has specifically focused on promoting interaction within the dyads of the following: (1) terminally ill adult children and their parents and (2) terminally ill parents and their adult children. A national Delphi study was conducted to provide appropriate recommendations for dyadic psychosocial support measures.

**Methods:**

Recommendations were formulated from qualitative interview data on the experiences and wishes of patients and family caregivers within these two dyads. Experts from palliative and hospice care providers rated the relevance and feasibility of 21 recommendations on two 4-point Likert-type scales, respectively. Additional suggestions for improvement were captured via free text fields. Individual items were considered consented when ≥ 80% of participants scored 1 (strongly agree) or 2 (somewhat agree) regarding both relevance and feasibility.

**Results:**

A total of 27 experts (35% response rate) completed two Delphi rounds. Following the first round, 13 recommendations were adjusted according to participants’ comments. After the second round, consensus was achieved for all 21 of the initially presented recommendations.

**Conclusion:**

The Delphi-consented recommendations for parents and adult children at the end of life provide the first guidance for hands-on dyadic psychosocial support measures for parent–adult child relationships, specifically. The next step could involve the structured implementation of the recommendations, accompanied by scientific research.

This study was registered on October 27, 2017, with the German Clinical Trials Register (DRKS00013206).

**Supplementary Information:**

The online version contains supplementary material available at 10.1007/s00520-021-06452-x.

## Introduction and background

In our aging society, the number of people facing terminal illness with a limited life span is increasing. Within families, parents are increasingly faced with the challenge of caring for an adult child with terminal illness; likewise, adult children are continuing to cope with the limited life span of their aging parents. In the context of terminal illness, both the family setting and the interaction between patients and relatives have a vital impact on the quality of life of patients and the burden experienced by their relatives. Furthermore, the quality of life of patients and caregivers is associated with the availability of psychosocial support; when the development or availability of such measures is lacking, needs are often unmet [1–8]. In general, patients and caregivers—as well as specific subsets of patients and caregivers—are likely to have very different psychosocial needs [6, 9]. For this reason, it is necessary to develop supportive interventions tailored to specific populations [1, 7].

Regarding the desired levels of support in managing end-of-life situations, both adult child caregivers and the parents of ill adult children seek information on the patient’s illness and perceive such information as helpful, because it enables them to take some control over the situation [10–13]. The term “caregiver” is used in this article in a broad sense, encompassing family members who provide practical and/or emotional support. The literature highlights the importance of family-specific patterns of communication [14]. Exchange with other caregivers [15], friends, and family members about feelings and events, as well as professional psychosocial support, have been shown to be valued by parent caregivers of adult children [10]. Research has also demonstrated the role of psychosocial support interventions in opening dialogue between terminally ill parents and their adult children [16]. Furthermore, the exchange of happy memories has been found to help patients find peace when nearing death [17–19]. Finally, one of the few studies on long-distance adult child caregivers for their dying parents demonstrated that feelings of guilt were powerful and needed to be addressed [20].

Several dyadic psychosocial interventions for patients with life-limiting illnesses and their caregivers have been developed [21–25]. Many of these interventions have involved patients’ significant others, to beneficial effect. For instance, Moon and Adams [26] showed, in their literature review, that relationship quality, well-being, and quality of life improved following a dyadic intervention for early dementia patients and their caregivers. Nezu et al. [27] also reported positive effects for a problem-solving therapy intervention for distressed adult cancer patients and their significant others, including improvements in their relationship, quality of life, and coping ability. However, none of these interventions specifically focused on promoting interaction within dyads of adult children and parents in end-of-life situations.

## Research question

The aim of the Delphi study was to provide practical recommendations for dyadic psychosocial support measures for the following: (1) terminally ill adult children and their parent caregivers and (2) terminally ill parents and their adult child caregivers. These recommendations were intended to support professionals and voluntary workers in inpatient and ambulatory hospice and palliative care providers.

## Methods

### Design

To develop and provide high-quality support for terminally ill and dying patients and their relatives, the specific needs of all parties must be known. The project “Dy@EoL—interaction at the end of life in dyads of parents and adult children” [28] formulated recommendations on the basis of empirical data on the experiences and desires of patients and family caregivers in each dyad.

**Fig. 1:**
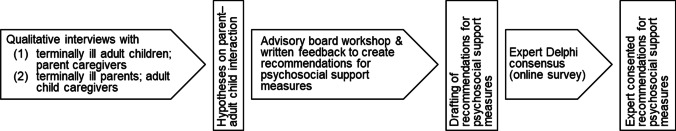
Study design

Three methodological steps were taken to formulate these recommendations (Fig. 1):

(I) Three core themes regarding parent–adult child interaction at the end of life emerged from the Dy@EoL qualitative interviews with parents and adult children in dyads 1 and 2 (February 2018 to November 2019): (1) relationship with the dyad partner [29], (2) communication and information [30], and (3) support and relief [31]. Individual hypotheses (*N* = 22) describing the interaction specifics of parents and adult children at the end of life were derived from these empirical data for the three subject areas.
(II) For the 22 individual hypotheses, the project team formulated recommendations for practical dyadic psychosocial support measures via written feedback and a workshop involving the project’s advisory board. The developed recommendations were pre-tested by one psychologist/palliative care researcher (S.S.; March to June 2020).
(III) The recommendations were reviewed using a national Delphi process (June to July 2020). The aim of the Delphi process was the following: (a) evaluate the individual recommendations according to their relevance and feasibility in everyday hospice and palliative care practice and (b) sharpen the wording. The Delphi technique was employed because it enables consensus to be achieved from a wide range of knowledgeable participants when face-to-face discussion is unfeasible [32–38]. In total, 21 recommendations were presented, of which (a) 5 specifically addressed dyad 1, (b) 11 specifically addressed dyad 2, and (c) 5 pertained to both dyads. In the Delphi survey, each recommendation was presented alongside its associated hypothesis, as recommendations can only be fully understood in the context of their underlying logic. Experts were invited by e-mail to participate in the survey. The Delphi survey was designed and administered anonymously online, using the software EvaSys V8.0 (Electric Paper Evaluationssysteme GmbH, Lüneburg, Germany). Experts rated the relevance and feasibility (with respect to the provision of care) of each recommendation on two 4-point Likert-type scales, respectively, ranging from 1 (strongly agree) to 4 (strongly disagree). Experts could also provide open comments and suggestions for improvement in a free text field connected to each recommendation.

### Participants

The advisory board consisted of 15 palliative care representatives from the fields of medicine, psycho(onco)logy, pastoral care, nursing, social work, and bereavement care, as well as hospice and palliative care networks. The invited experts (*n* = 77) for the Delphi study comprised advisory board members, all tenured professors for palliative medicine in Germany, and further representatives from palliative and/or hospice care, including researchers with expertise in parent–adult child interaction at the end of life. All experts were informed of the nature and aim of the study in an e-mail that included the link to the Delphi survey. The experts participated voluntarily and were not compensated for their participation; however, they were invited to participate in a draw of three 50€ vouchers for an online store.

### Data processing and analyses

All free text comments collected from the Delphi study were independently analyzed by two researchers (F.A.H., L.G.) and used to refine the individual recommendations. Quantitative data from the survey were exported to Microsoft Excel 2010 and IBM SPSS Statistics 26 (SPSS Inc., Chicago, IL, USA), for the purpose of descriptive analysis. The Guidance on Conducting and REporting DElphi Studies (CREDES) in palliative care checklist [39] was used to ensure comprehensive reporting of the Delphi study.

In accordance with similar studies [36, 40, 41], each recommendation was considered consented when ≥ 80% of participants scored the item 1 (strongly agree) or 2 (somewhat agree) for both relevance and feasibility. Although the Delphi method does not require items that achieve a high level of agreement in the first round to be added to the second round [42], in the present study, the authors (F.A.H., L.G.) included some items with an initial high rating in the second round because the experts provided valuable free text comments that the authors wished to incorporate.

## Results

### Sample characteristics

For Delphi round 1, 77 experts were invited to participate, and 43% took part (*n* = 33/77). These 33 respondents were also asked to participate in the second round. In round 2, 82% of the invited participants (*n* = 27/33) completed the survey. Thus, the final response rate was 35% (*n* = 27/77) (see Table 1 for participants’ socio-demographic characteristics).

**Table 1:** Sample socio-demographic characteristics of Delphi study rounds 1 and 2 participants

		Round 1	Round 2
No. participants		33	27
Age	Mean; SD	51.6 ± 10.8	49.3 ± 11.0
	Range (years)	34–70	31–70
	Missing	5	3
Sex	Female	75.8%	77.8%
	Male	24.2%	22.2%
Profession	Physician (hospital)	7	6
	Physician (resident)	1	1
	Nursing	1	1
	Psycho(onco)logy	13	11
	Social work/pedagogy	8	5
	Pastoral care	1	1
	Other†	2	2
Field of work	Palliative care unit/palliative consulting service (hospital)	18	12
	Specialized palliative home care team	1	1
	Hospice	2	2
	Hospice home care service provider	2	2
	Practice (resident)	3	2
	Research	3	5
	Other‡	4	3
Work experience	Mean; SD	20.0 ± 12.4	19.6 ± 12.4
	Range (years)	2–40	2–44

*SD* standard deviation

^†^Hospice coordination, sociology

^‡^Intensive care and normal units; coordination and organizational development; political advisory work

### Consensus on recommendations

#### Delphi round 1

In Delphi round 1, 19 out of 21 (90.5%) of the presented recommendations achieved consensus with respect to both assessment criteria. The panelists disagreed on 2 (9.5%) items, and these items were adjusted for the second Delphi round according to participants’ free text comments (see Table 2).

**Table 2:** Core themes and number of Delphi recommendations

	Core theme	Initial number of items	Delphi round 1			Delphi round 2			Final number of items
			Unconsented	Consented	Consented	Unconsented	Consented	Consented	
			Adapted	Adapted		Adapted	Adapted		
1	Relationship with dyad partner	6	1	5	0	0	0	6	6
2	Communication and information	6	1	4	1	0	0	5	6
3	Support and relief	9	0	2	7	0	0	2	9
	Sum	21	2	11	8	0	0	13	21

For the first core theme, “relationship with dyad partner,” all recommendations except for R-1.5 achieved consensus in both evaluation criteria (see Table 3 on levels of agreement). R-1.5 achieved consensus with respect to relevance for everyday practice, but only 75.8% consensus in terms of feasibility. Participant feedback indicated concern that the actors who would be tasked with realizing this recommendation were not specified. Furthermore, the limited time involved in end-of-life situations was seen to hinder the suggested biography work. Finally, participants commented that they were missing information on how the ill parent and adult child caregiver would be supported in allowing and voicing accusations from and towards the dyadic other.

**Table 3:** Level of agreement on relevance and feasibility for Delphi statements

Recommendation	Consented in round	Dyad	Agreement relevance	Agreement feasibility	*n*
			**%**	**%**	
1st core theme: relationship with dyad partner					
R-1.1	2 (1)	1 + 2	100	96.3	27
R-1.2	2 (1)	1 + 2	100	100	27
R-1.3	2 (1)	1	100	96.3	27
R-1.4	2 (1)	1	96.3	85.2	27
R-1.5	2	2	100	92.6	27
R-1.6	2 (1)	2	100	96.3	27
2nd core theme: communication and information					
R-2.1	2 (1)	1 + 2	96.3	92.6	27
R-2.2	2 (1)	1 + 2	100	92.6	27
R-2.3	1	1	93.9	87.9	33
R-2.4	2(1)	2	96.3	96.3	27
R-2.5	2	2	96.3	92.6	27
R-2.6	2 (1)	2	96.3	96.3	33
3rd core theme: support and relief					
R-3.1	1	1 + 2	93.9	87.9	33
R-3.2	1	1	93.9	90.9	33
R-3.3	1	1	93.9	97.0	33
R-3.4	1	2	97.0	97.0	33
R-3.5	1	2	97.0	87.9	33
R-3.6	1	2	93.9	97.0	33
R-3.7	2 (1)	2	100	92.6	27
R-3.8	1	2	97.0	90.9	33
R-3.9	2 (1)	2	96.3	92.6	27

Dyad 1: terminally ill adult children and parents; dyad 2: terminally ill parents and adult children

With respect to the second core theme, “communication and information,” one (R-2.5) of the six recommendations only achieved 75.8% approval with respect to feasibility and, thus, did not achieve consensus in the first round. Participants endorsed the inclusion of an explanation that psychosocial support would only be offered when family communication patterns were perceived as burdensome and the patient and/or caregiver expressed a need for change. Depending on the situation, only one dyad partner or both dyad partners would be offered support.

All recommendations for the third core theme, “support and relief,” achieved consensus with respect to both evaluation criteria.

In addition to the unconsented recommendations R-1.5 and R-2.5, 11 (R-1.1, R-1.2. R-1.3, R-1.4, R-1.6, R-2.1, R-2.2, R.2.4, R-2.6, R-3.7, R-3.9) of the 19 consented recommendations were adapted for the second Delphi round, in order to incorporate participants’ valuable feedback for improvement.

Participants’ suggestions regarding the first core theme, “relationship with dyad partner” (R-1.1, R-1.2. R-1.3, R-1.4, R-1.6), primarily pertained to exploring the experience of losing a loved one, non-judgmental responding to patients’ and family caregivers’ perceptions of their relationship, and the potential opportunities involved in exploring role reversal and individual needs.

Regarding the second core theme, “communication and information” (R-2.2, R.2.4, R-2.6), participants’ remarks emphasized their need for clarification on the professions of those who would be offering support to patients/caregivers, particularly with respect to supporting patients/caregivers in accepting that communication may change in the terminal illness situation and that, at some point, patients may not wish to talk about dying and death.

Only two recommendations pertaining to the third core theme, “support and relief” (R-3.7, R-3.9), were included in the second Delphi round. Participants’ strongest suggestions were to support caregiving adult children in developing different attitudes towards their feelings of guilt around their limited ability to care for their ill parents and to elicit additional resources to help patients and caregivers.

Some comments cut across the individual recommendations and were incorporated into an introductory text that framed the recommendations. These comments concerned the importance of considering individual differences between patients (with respect to, e.g., their expected remaining lifetime and perceptions of what is burdensome), the application of the recommendations only when patients/caregivers expressed a need for action, the framework conditions in which the recommendations would be applied (e.g. ambulatory vs. inpatient settings), and clarification that the recommendations were directed at psychosocial professionals, palliative care teams, and family doctors.

#### Delphi round 2

Thirteen recommendations (R-1.1, R-1.2, R-1.3, R-1.4, R-1.5, R-1.6, R-2.1, R-2.2, R.2.4, R-2.5, R-2.6, R-3.7, R-3.9) were modified according to participants’ comments (see Appendix 1 for the consented Delphi statements) and presented in the second Delphi round. All of these recommendations achieved consensus, with an average agreement rate of 98% and 94% regarding relevance and feasibility, respectively. Hence, consensus on all 21 recommendations was achieved within the two-round Delphi process; none of the initial recommendations was dropped.

## Discussion

In this Delphi study, we consented 21 recommendations for psychosocial measures to support interaction within two adult child–parent dyads. The majority of the recommendations were immediately consented in the first round, and all achieved ≥ 80% agreement regarding their relevance. We suggest that this result is due to the methodological approach of the Dy@EoL project: hypotheses were deduced from interviews with affected parents and adult children in both dyads. In addition, the recommendations were developed in collaboration with an advisory board and grounded in participants’ clinical practice. Several lines of evidence have suggested that fit, relevance, and quality of research can be enhanced by involving those for whom the support measures are designed and those who will be tasked with their delivery [43–45].

The agreement on feasibility was relatively lower, with two recommendations not consented in the first round but achieving consensus in the second. The partial lack of specification of the professional groups (e.g., palliative care teams, psycho-oncologists, or family doctors) at whom the recommendations were directed and who would administer the recommended measures as well as the limited time involved in end-of-life situations were perceived as limiting factors for implementation in palliative and hospice care practice. All other recommendations gained approval in the first round. We argue that this is due to the fact that our recommendations had a “modest aim” [1]: they did not describe a comprehensive intervention program but instead represented practical tips for multi-professional palliative care teams to better support adult child–parent dyads at the end of life. The Delphi survey was necessary to close the remaining gap between research and practice. Overall, research and practice dovetailed in the project, and apparently, this had a positive effect on the consensus-building process.

## Strengths and limitations

Current psychosocial support measures represent an important aspect of hospice and palliative care. The recommendations developed in the present study provide additional guidance for the specific situations faced by parents and adult children at the end of life. These recommendations are not to be understood as guidelines, but rather practical, hands-on support. The recommendations were specifically developed for application in ambulatory and inpatient German healthcare contexts. This may limit the generalizability of the results to other countries. Furthermore, the application and scope of the recommendations depend on the life expectancy of the patient and the accompanying caregiving period. In the context of longer caregiving periods, it may be useful to repeatedly assess patients’ and relatives’ feelings of burden and support needs. A further limiting factor of the present study is the relatively low response rate (35%). Nevertheless, participants’ clinical expertise, long-standing experience, and multi-professionalism represent an absolute strength. The number of experts from psychosocial professions participating in the Delphi survey was particularly high. These participants may feel more addressed than other experts as recommendations explicitly present psychosocial support. Reasons for non-participation and dropout could not be gleaned.

## Conclusions

Interview data from the Dy@EoL study supported previous findings on support experiences and needs in end-of-life situations within dyads of parents and adult children [10, 16–20] and revealed further dyad-specific experiences and needs. The consented recommendations in the described Delphi study present the first practical support grounded in empirical data collected from parents and adult children within both patient–caregiver dyads for managing their psychosocial needs. The recommendations provide useful guidance for the development of manifold dyadic psychosocial support measures targeting parent–child relationships, specifically. The German recommendations were published as a pocket-sized brochure, in cooperation with the German Association for Palliative Medicine [46], and are freely accessible online for application in healthcare practice. The next step could involve the structured implementation of the recommendations, accompanied by scientific research.

## Supplementary Information

Below is the link to the electronic supplementary material.

Supplementary file1 (PDF 418 KB)

Supplementary file2 (PDF 206 KB)

## Data Availability

The datasets analyzed in this study are available from the corresponding author upon reasonable request.
