# Supplementary material for: Consensus-based recommendations for psychosocial support measures for parents and adult children at the end of life: results of a Delphi study in Germany
Source: Support Care Cancer. 2021 Aug 7;30(1):669–76. doi: 10.1007/s00520-021-06452-x (PMC8636430; doi:10.1007/s00520-021-06452-x)
Supplement: Supplementary file 1 — Supplementary file1 (PDF 418 KB) [file 520_2021_6452_MOESM1_ESM.pdf]

No.	Dyad	Hypothesis	Delphi statement
			**1st core theme: relationship with dyad partner**
R-1.1	1+2	The loss of the patient is experienced as existential.	*Without judgment (i.e., without implicitly weighting the loss), the palliative care team or family doctor allows the caregiver space to discuss possible concerns related to the loss of the patient.* The family doctor *or* clinic offers active psychological support to the caregiver if s/he has existential fears and needs (and potentially also provides flyers about counseling) *and* initiates the process if the caregiver *wants support* but is not able to initiate it. In a *psycho(onco)logical* conversation, the caregiver *receives* confirmation of his/her situation and any measures deemed helpful to manage it. *The conversation explores whether the caregiver has already experienced any loss of a loved one and how s/he previously dealt with it.* Explore whether the patient or caregiver knows other people who have had similar experiences *and whether they* might be able to help the patient or caregiver deal with the situation. *Introduce* offers for mourning and self-help groups.
R-1.2	1+2	The illness situation and resulting dependencies are perceived as role reversal or contrary to the “natural order.”	In a *psycho(onco)logical conversation*, *meet* the perceived role reversal *in a non-judgmental way*, name it, and address that the feeling of role reversal is common. Explore whether the role reversal is subjectively perceived as burdensome *and to what extent it can represent an opportunity*. If appropriate, initiate support measures (e.g. delegating tasks to other relatives or friends; offering support from a hospice service).
R-1.3	1	The parent wants to be closer to the patient, whereas the patient wants more freedom.	Offer a psycho*(onco)*logical conversation to the patient and parent to support them in openly communicating to the dyad partner their respective understanding of and need for autonomy *in relation to the current situation and the care provision*. *In advance, the palliative care team or family doctor clarifies with the patient whether s/he is willing to have a joint conversation with the parent, if the parent also agrees. If appropriate, offer patient assistance in verbalizing to the parent the need for rest.* Discuss together, *in a psycho(onco)logical conversation,* what can be shared *and what connects, underlining that this is not a general criticism or rejection of the parent*.
R-1.4	1	The patient perceives a strong burden or excessive demand on the parent and feels the need to build up the parent, so that the relationship is experienced by the patient as tense or lacking understanding.	*The palliative care team or family doctor determines whether an individual discussion or clarifying family discussion is desired and, if so, whether this might have a relieving effect. If appropriate,* offer a family discussion *with psychologically trained staff* to make different needs transparent and to articulate expectations and wishes. In the conversation, reduce possible tensions between dyad partners by allowing any emotions experienced in the situation to be expressed and by addressing fears and concerns. *Offer* other support measures (e.g. socio-legal counseling*, referral to self-help groups) to* distribute the perceived burden.

R-1.5	2	On the one hand, the patient and the adult child share childhood memories; on the other hand, the adult child gets to know the patient from a new side.	Create space for sharing *(childhood)* memories *through psychology/pastoral care/volunteer accompaniment*. *Offer* psychological support if the sharing of *(childhood)* memories or the experience of getting to know the parent from a new side is perceived as burdensome or gives rise to historical conflicts. *If appropriate,* encourage a change of perspective to promote understanding and trust *in the current situation*. *Facilitate psycho(onco)logical support for the patient and adult child, helping them to accept and express reproach.*
R-1.6	2	The patient and the adult child experience a new or re-established intensity of the relationship in the current illness situation.	In conversation with the palliative care team *or family doctor,* clarify the extent to which intensity is appreciated or perceived as burdensome. If desired, enable intensive encounters (e.g. *joint activity of the dyad partners; if possible,* by admitting the adult child as an inpatient). *Offer the adult child socio-legal counseling on topics including* caregiver and job leave. Offer support from hospice or social services.
			**2nd core theme: communication and information**
R-2.1	1+2	The parent or adult child wants to talk to the patient about the patient's passing and things that will need to be settled after the death.	*The palliative care team or family doctor elicits whether the patient is ready for a joint conversation with the caregiver about dying and anything that needs to be settled after death. If desired by the dyad, the palliative care team or family doctor* promotes intra-family communication by encouraging open exchange and mediating the conversation between patient and caregiver. Offer socio-legal and psychological counseling about what might be settled before the patient dies and how the process of clarifying anything that needs to be settled after death can be relieving for both dyad partners. Offer a separate conversation with the caregiver if the patient does not want to talk about *dying* and death. Broach concerns and fears with the caregiver and, when appropriate, provide flyers on advance directives and health care proxies, and extend offers for further conversation *(and, if necessary, bureaucratic procedures)*.
R-2.2	1+2	The patient does not talk (or avoids talking) about his/her illness or death, also to spare the parent or adult child.	*The palliative care team or family doctor accepts that the patient might avoid the topics of death/dying and not wish to talk about them.* Elicit whether silence or talking/knowing is more stressful. *If the patient suffers from his/her inability or unwillingness to communicate,* offer communication support (from physicians, psychologists or the palliative care team) to help the patient and caregiver openly express concerns. Try to build understanding between dyad partners for possibly divergent communication needs *and encourage a change in perspective*. Respect when the patient does not want to engage in a family conversation or does not wish to talk to the caregiver about his/her situation. If verbal exchange is perceived as too burdensome, offer alternatives (e.g. a written formulation *or audio recording*). If appropriate, offer the caregiver a separate discussion or psychoeducation on diversity of communication at the end of life.

R-2.3	1	The patient seeks open communication about his/her illness with the parent or wants the parent to receive information about the illness situation.	Offer the patient and parent support from the palliative care team (e.g. joint consultation with the physician). If applicable, find out why the parent does not want to talk to the patient about his/her illness. Professional caregivers can try to encourage the family caregiver to talk to the patient by intercepting possible fears and worries, but also addressing the patient's and family caregiver's right to remain uninformed.
R-2.4	2	It is important for the patient to discuss with the adult child arrangements concerning death, burial or inheritance, also in order to prepare the adult child.	*If requested by the patient, the palliative care team or family doctor* addresses the patient's needs with the adult child *or supports the patient in doing this him/herself*. Point out the potentially relieving function for the adult child to be able to act in the patient's interest. When desired, the palliative care team/psychologist/social worker facilitates an open conversation between the patient and the adult child and offers advice on other (e.g. socio-legal) concerns that might be important from a professional point of view. *If the adult child is not willing/able to discuss or settle everything in the current situation, offer support, if appropriate.*
R-2.5	2	Established family communication patterns influence whether the patient and adult child can communicate openly in the current illness situation.	*If the existing family communication pattern is perceived by the patient and adult child as burdensome and there is a desire to change it, offer a (family therapy) conversation with the palliative care team/family doctor/*psycho(onco)logy. *Create a safe space for open exchange between the patient and adult child. If applicable, mediate communication between the patient and adult child. If only one dyad partner experiences the current communication pattern as burdensome, elicit whether a one-on-one conversation might be relieving.* Since established family communication patterns are difficult to change in the short term, offer patient and caregiver alternatives (e.g. writing a letter).
R-2.6	2	Communication has changed with the illness situation; this is experienced as positive, but simultaneously artificial relative to previous communication patterns.	*If the patient or adult child perceives the change in communication as burdensome, offer support* from the palliative care team/*family doctor/*psycho(onco)logy in dealing with the change and, *if applicable,* discuss possible reasons for the change with the patient or adult child. *If necessary, support* the patient or adult child in talking about the perceived emotional burden resulting from the change in communication and elicit transparency as to which aspects of the change are perceived as positive and which are perceived as "strange." *Support the patient or adult child in accepting the change in communication.*

			**3rd core theme: support and relief**
R-3.1	1+2	The patient was the primary caregiver for the parent or adult child prior to the current illness situation, and wants to continue to support the family member; he or she is concerned about leaving them behind.	Elicit, together with the patient and caregiver, the extent to which the caregiver is dependent on the patient. If appropriate, support the patient in compiling useful information and recommendations for the time after the patient's death. Offer a clarifying conversation about what additional resources are available to the caregiver and activate the appropriate resources. Offer professional support (e.g. social work) if the situation results in organizational or financial problems for the caregiver. Create confidence and trust that the caregiver can develop his/her own resources to cope. Allow for the experiences of pain and regret.
R-3.2	1	Because of the special qualities of the parent–child relationship, the parent provides care more naturally and accurately than other relatives.	Address role advantages and disadvantages in conversation with the palliative care team/psycho(onco)logy. Explore who fulfills which support role for the patient. Encourage the parent to practice self-care. Point out the advantages of distributing caregiving responsibilities among others in order to maintain the parent's strength.
R-3.3	1	The patient perceives limits in the support provided by the parent or wants to spare the parent because the parent has little endurance due to advanced age; supporting the patient is exhausting for the parent due to age and goes beyond the parent's own strength.	Clarify, through conversation, which tasks the parent can and wants to take on. Encourage the parent to take responsibility for him/herself and show how this will benefit the patient. Ensure a realistic (self-)assessment of the patient's strengths in order to offer suitable help and relief (e.g. professional ambulatory or inpatient care). If appropriate, integrate other professional service providers or relatives to ensure comprehensive care.

R-3.4	2	The physical care required goes beyond that which was provided in the previous parent–child relationship; this can create new intimacy, but the patient or adult child may also reject it.	Clarify, in conversation with the palliative care team/social work, the extent to which physical care for the patient is perceived as a support or burden. To give the adult child space to discuss physical care, explicitly address that nursing tasks can generate feelings of aversion and are sometimes taken on, in part, due to a perceived social obligation. Determine with the adult child which care activities are "authentically" offered and which trigger inner rejection. Discuss the individual limits of the dyad partners and the danger of losing previous roles when the adult child takes over care, as well as feasible forms of support. Offer solutions for tasks that cannot be fulfilled, such as socio-legal advice on care levels, care leave and the provision of professional support services (e.g. from a home care team). Provide practical guidance from an experienced professional nurse to adult children who want to perform nursing tasks.
R-3.5	2	The adult child can only provide limited support to the patient due to (spatial) distance. The adult child negotiates distance differently (e.g. through sustained face-to-face presence, by coordinating visits with siblings, or by making him/herself available at a distance at all times).	Explore, in a family discussion, the patient's needs and the adult child's resources. In the event that criticisms or feelings of guilt are acknowledged, encourage the adult child to maintain his/her daily routine, even in the present crisis situation. Assist the adult child in developing routines that are relieving and reassuring. Offer socio-legal advice on caregiver leave and special leave, and discuss alternative support and accommodation options (e.g. support from other relatives or friends; home care services and hospice services; or, if applicable, specialized palliative home care, 24-hour emergency support and hospice care). In the event of spatial distance, discuss the possibilities of digital communication. Where appropriate, determine whether the patient might explore moving closer to the adult child.
R-3.6	2	The patient appreciates the adult child's commitment but does not want to overburden him/her and is understanding of the adult child's other responsibilities.	In a conversation accompanied by a psychological team, encourage open communication about the actual burden and clarify with the adult child whether s/he is truly as burdened as the patient perceives him/her to be. Determine the needs of the adult child with regard to the frequency of contact. Offer further support options (e.g. hospice care, specialized palliative home care and self-help groups for relatives).
R-3.7	2	The adult child feels guilty if he/she can only provide limited support to the patient.	In a *psycho(onco)logical* conversation, give the adult child space to address his/her fears and feelings of guilt, as well as possible criticisms, *and embed them in the current situation*. *Support the adult child in developing a different attitude towards his/her feelings of guilt. If desired by the adult child, address the patient's feelings of guilt in a psycho(onco)logical conversation.* Encourage *the adult child* to seek socio-legal advice and a home care service. Advise the adult child that exchange with other affected persons or self-help groups can also be supportive.

R-3.8	2	The adult child's desires take a back seat in the illness situation.	In conversation with the palliative care team, emphasize the importance of self-care to the adult child, and possibly put into perspective overly high expectations that the adult child may have of him/herself. Work out with the adult child what will help him/her maintain his/her strength. Activate resources (e.g. own well-being and sources of strength) and encourage the adult child to create free space or take time out. Encourage exchange with others (e.g. in self-help groups).
R-3.9	2	The patient worries about the future of the adult child after his/her death.	*The palliative care team or family doctor gives the patient the opportunity to address concerns. Clarify whether worries have rational reasons and what, if any, practical arrangements can be made to alleviate them. If appropriate,* offer psychological support and express in a psychological discussion that the patient's worries are understandable, particularly as an expression of the parent–child bond; also strengthen confidence in the adult child's abilities. *Offer the patient and adult child a conversation to elicit what additional resources the adult child might have available and activate the appropriate resources.*

Appendix: Consented Delphi statements with underlying hypotheses.

Legend: Dyad 1: terminally ill adult children and parents; Dyad 2: terminally ill parents and adult children. Italics indicate modifications following round 1.
